# Fear of childbirth and sleep quality among pregnant women: a generalized additive model and moderated mediation analysis

**DOI:** 10.1186/s12888-023-05435-y

**Published:** 2023-12-11

**Authors:** Xiaoxiao Mei, Ping Du, Yan Li, Ranran Mei, Xinqin Wang, Qianwen Chen, Zengjie Ye

**Affiliations:** 1 School of Nursing, Hong Kong Polytechnic University, Hong Kong, China; 2 School of Nursing, Guangzhou University of Chinese Medicine, Guangzhou, China; 3 Breast Oncology Department, Guangzhou Medical University Cancer Hospital, Guangzhou, China; 4 School of Nursing, Guangzhou Medical University, Guangzhou, China

**Keywords:** Pregnant women, Fear of childbirth, Psychological distress, Sleep quality

## Abstract

**Objectives:**

This study aims to examine the associations among fear of childbirth, psychological distress, resilience, and sleep quality among Chinese pregnant women.

**Methods:**

A cross-sectional survey was carried out between January 2022 to March 2022 among pregnant women who met the inclusion criteria and sought healthcare services at The First Affiliated Hospital of Guangzhou University of Chinese Medicine in Guangdong Province, Southern China. Data was collected using a structured questionnaire that included sociodemographic characteristics, childbirth attitudes questionnaires (CAQ), hospital anxiety and depression scale (HADS), Connor-Davidson resilience scale (CD-RISC), and Pittsburgh sleep quality index (PSQI). A generalized additive model and moderated mediation analysis were employed for data analysis.

**Results:**

A non-linear and negative association between fear of childbirth and sleep quality was found in the second trimester and antenatal period. Psychological distress significantly mediated the relationship between fear of childbirth and sleep quality (first trimester: *β* = 0.044, *95%CI*:0.022–0.071; second trimester: *β* = 0.029, *95%CI*:0.009–0.056; third trimester: *β* = 0.064, *95%CI*:0.046–0.088; antenatal period: *β* = 0.050, *95%CI*:0.037–0.063). The moderating role of resilience between fear of childbirth and sleep quality was significant (second trimester: *β*=-0.006, *95%CI*:-0.012–0.001, *P* = 0.025; antenatal period: *β*=-0.004, *95%CI*:-0.007–-0.001, *P* = 0.014), as well as between fear of childbirth and psychological distress (first trimester: *β*=-0.016, *95%CI*:-0.026–-0.005, *P* = 0.004; antenatal period: *β*=-0.005, *95%CI*:-0.009–-0.001, *P* = 0.014).

**Conclusions:**

Fear of childbirth, psychological distress, and resilience are three important factors affecting sleep quality in Chinese pregnant women.

**Supplementary Information:**

The online version contains supplementary material available at 10.1186/s12888-023-05435-y.

## Introduction

Pregnancy is a critical period marked by significant physiological and psychological changes that can impact sleep quality and mental health [1, 2]. Sleep quality is often used to assess sleep continuity, including sleep latency, sleep efficiency, sleep duration, and waking after falling asleep [3]. Poor sleep quality during pregnancy has been confirmed as a risk factor for pregnancy-related complications such as gestational diabetes and premature birth [4, 5]. In addition, Vizzini et al. conducted a birth cohort study that revealed a significant association between maternal sleep disturbances during pregnancy and the manifestation of ADHD symptoms in preschool-aged children [6]. Thus, it is essential to investigate factors that influence sleep quality in pregnant women.

One contributing factor is the phenomenon known as fear of childbirth, which refers to a complex feeling of distress before childbirth and commonly experienced by pregnant women, with symptoms of worry, extreme anxiety, and even the desire to avoid childbirth [7]. The prevalence varies across countries, with about 20% of pregnant women experiencing this fear in Sweden and Italy [8, 9], while a recent survey in China reported a prevalence of 67.1% [10]. This fear has been linked to adverse outcomes, including the progression of birth and an increased likelihood of elective cesarean Sects [11, 12]. In extreme cases, they will choose to terminate their pregnancy [13]. Moreover, studies have shown a positive relationship between fear of childbirth and sleep disturbance or disorders [14, 15].

In addition, psychological distress may also influence sleep quality [16, 17], and can be exacerbated by the presence of fear of childbirth [18, 19]. However, no previous research has investigated the mediating role of psychological distress in the relationship between fear of childbirth and sleep quality. Moreover, research has consistently shown that pregnant women with high resilience exhibit better-coping abilities in managing childbirth-related concerns and report lower levels of fear of childbirth [10, 20]. Furthermore, resilience has been confirmed as a crucial protective factor for mental health and better sleep quality among pregnant women [18, 21–23]. Thus, resilience may have an important role in the relationships among fear of childbirth, psychological distress, and sleep quality.

What’s more, research has shown that fear of childbirth, psychological state, and sleep quality are dynamic and change/fluctuate across trimesters [15, 24, 25]. Nevertheless, to date, no studies have employed stratified analyses by trimester to explore the relationship among these factors at different stages of pregnancy. By conducting stratified analyses based on trimesters of gestation, critical periods for intervention can be identified, and a comprehensive understanding of the complex relationships among these factors will be achieved. Given the existing knowledge gaps, this study aims to explore the association between fear of childbirth and sleep quality, considering the potential mediating role of psychological distress and the moderating role of resilience. We hypothesize the following:

H1: Fear of childbirth is a significant predictor of sleep quality across different trimesters and throughout the antenatal period.

H2: The relationship between fear of childbirth and sleep quality is non-linear across different trimesters and throughout the antenatal period.

H3: Psychological distress may mediate the relationship between fear of childbirth and sleep quality at different trimesters and throughout the antenatal period.

H4: Resilience may moderate the relationship between fear of childbirth, psychological distress, and sleep quality at different trimesters and throughout the antenatal period.

## Methods

### Design and participants

This study was conducted from January 2022 to March 2022 at the First Affiliated Hospital of Guangzhou University of Chinese Medicine in Guangdong Province, southern China. We employed a convenience sampling method to collect data from pregnant women attending regular prenatal check-ups at the hospital. The target population eligible to participate in this study should meet the following inclusion criteria: (1) be at least 20 years old (legal marriage age for Chinese women is 20); (2) have a confirmed pregnancy; (3) be able to communicate fluently in Mandarin. Pregnant women with diagnosed mental illness or a history of mental health illness were excluded from the study. The questionnaires were independently completed by recruited participants and collected by a fixed team of three trained enumerators to ensure data quality. To estimate the minimum sample size required for detecting a significant moderated mediation effect, we utilized the R-based “pwr” package. Based on the assumptions of a mediation effect of 0.05, a moderator-dependent variable relationship of 0.3, a mediator-dependent variable relationship of 0.4, an alpha level of 0.05, and a desired statistical power of 0.8, we determined that at least 503 participants were needed for the study.

### Instruments

#### Demographics

Based on prior research [13, 26], this study collected demographics, including age, academic degree, employment, income, and place of residence, as well as clinical information on weeks of pregnancy.

#### Childbirth attitudes questionnaires (CAQ)

The CAQ was developed by Tanglakmankhong et al [27], and its Chinese version was validated by Zhou et al [28]. It consisted of 16 items, ranging from 16 to 64, with higher scores indicating higher levels of fear of childbirth. The Cronbach’s α for CAQ in the current study was 0.945.

#### Hospital anxiety and depression scale (HADS)

The HADS was developed by Zigmond and Snaith [29], and validated in Chinese by Leung et al [30]. It has 14 items and ranges from 0 to 42, with higher scores indicating higher levels of anxiety and depression (psychological distress). In this study, Cronbach’s α was 0.745 for the anxiety domain, 0.700 for the depression domain, and 0.820 for the total scale.

#### 10-item connor-davidson resilience scale (CD-RISC-10)

The CD-RISC-10 was validated by Campbell-Sills and Stein [31]. It ranges from 0 to 40, with higher scores indicating higher levels of resilience. Ye validated the Chinese version [32], and it was previously used in our previous research [18]. The Cronbach’s α in this study was 0.915.

#### Pittsburgh sleep quality index (PSQI)

The PSQI was developed by Buysse et al [33], and its Chinese version has been commonly used to evaluate sleep quality among pregnant women in China [26, 34]. It has seven domains, including sleep duration, sleep latency, sleep disturbances, subjective sleep quality, use of sleep medication, habitual sleep efficiency, and daytime dysfunction. The total score ranges from 0 to 21, with higher scores meaning poorer sleep quality. The Cronbach’s α was 0.715 in the present study.

### Data analysis

First, independent samples t-test and one-way ANOVA were used to investigate differences in demographic factors of participants’ sleep quality (continuous variable). Demographic factors that exhibited statistical significance for each pregnancy were incorporated as covariates in subsequent mediated moderation models. Then, based on a cut-off of 7 [35], sleep quality was categorized into binary data (poor sleep was coded as 1 while good sleep was coded as 0). The relationship between fear of childbirth and sleep quality (category variable) was analyzed.

Second, the potential non-linear correlation between fear of childbirth and sleep quality in different trimesters of pregnancy was estimated by a generalized additive model [36].

Third, Pearson’s correlation analysis was conducted to ascertain the relationships among fear of childbirth, psychological distress, resilience, and sleep quality.

Fourth, the potential presence of common method variance was estimated by Harman’s one-factor model [37]. Subsequently, a comprehensive statistical approach was employed to examine both mediation and moderation effects, taking into account different covariates (confounders) in each trimester. The mediating role of psychological distress between fear of childbirth and sleep quality was investigated separately for each trimester of pregnancy. To achieve this, we utilized the PROCESS macro (model 4) for SPSS, developed by Hayes [38]. Then, model 5, model 7, and model 14 from the PROCESS macro were used to examine the moderating role of resilience on the direct relationship between fear of childbirth and sleep quality, as well as the indirect relationship through psychological distress.

Fifth, the Johnson-Neyman test was used to further probe the interaction pattern and identify statistically significant cut-off values for the moderating effect [39].

All statistical analyses were conducted using SPSS (version 26.0) and Empower Stats (version 2.2).

### Ethical considerations

This study was approved by the ethics review committee of the participating hospital (No: K-2022-024) and was part of Be Resilient to Postpartum Depression (BRPD, Registration number: ChiCTR2100048465). Written consent was obtained before a formal investigation. Besides, the participants were informed that their data would be kept private and used anonymously for academic research.

## Results

### Sample characteristics

A total of 768 pregnant women were included in this study after excluding 48 individuals due to missing questionnaires, resulting in a participation rate of 94.1%. The mean age of the included pregnant women was 29.26 years (SD = 4.57), and one-third (35.2%) of the women had tertiary education. More than half (51.6%) were in the third trimester of pregnancy. Other details are demonstrated in Table 1.

**Table 1:** Demographic and relevant characteristics differences in the score of sleep quality (continuous variable)

Variable	Model 1 (First trimester)			Model 2 (Second trimester)			Model 3 (Third trimester)				Model 4 (Antenatal period)			
	M ± SD	Number (22.5%)	*P* value	M ± SD	Number (25.9%)	*P* value	M ± SD	Number (51.6%)	*P* value	M ± SD		Number (100.0%)	*P* value	
Age	6.31 ± 2.88	28.62 (4.93)	0.260	6.20 ± 3.10	29.41 (4.59)	0.703	7.11 ± 3.19	29.46 (4.39)	0.004	6.69 ± 3.13		29.26 (4.57)	0.012	
Academic degree, n(%)			0.092			0.293			0.019				0.071	
High school or less	6.02 ± 3.13	58 (33.5%)		6.60 ± 3.33	73 (36.7%)		6.50 ± 2.84	136 (34.3%)		6.42 ± 3.04		267 (34.8%)		
Junior college degree	6.98 ± 2.88	58 (33.5%)		6.13 ± 2.97	75 (37.7%)		7.54 ± 3.21	137 (34.6%)		7.03 ± 3.12		270 (35.2%)		
Bachelor or above	5.93 ± 2.52	57 (33.0%)		5.73 ± 2.93	51 (25.6%)		7.29 ± 3.47	123 (31.1%)		6.61 ± 3.20		231 (30.0%)		
Employment, n(%)			0.844			0.010			0.990				0.141	
Yes	6.28 ± 2.94	120 (69.4%)		5.86 ± 2.85	145 (72.9%)		7.11 ± 3.31	270 (68.2%)		6.58 ± 3.16		535 (69.7%)		
No	6.38 ± 2.75	53 (30.6%)		7.13 ± 3.55	54 (27.1%)		7.10 ± 2.93	126 (31.8%)		6.94 ± 3.05		233 (30.3%)		
Monthly average household income, n(%)			0.333			0.011			0.075				0.584	
≤ 4000 RMB	6.70 ± 3.08	40 (23.1%)		7.28 ± 3.56	51 (25.6%)		6.59 ± 2.89	92 (23.2%)		6.80 ± 3.13		183 (23.8%)		
> 4000 RMB	6.20 ± 2.82	133 (76.9%)		5.83 ± 2.85	148 (74.4%)		7.26 ± 3.26	304 (76.8%)		6.66 ± 3.13		585 (76.2%)		
Place of residence, n(%)			0.599			0.953			0.629				0.942	
City or town	6.19 ± 2.90	80 (46.2%)		6.19 ± 3.13	81 (40.7%)		7.20 ± 3.18	168 (42.4%)		6.70 ± 3.13		329 (42.8%)		
Countryside	6.42 ± 2.88	93 (53.8%)		6.21 ± 3.09	118 (59.3%)		7.04 ± 3.20	228 (57.6%)		6.69 ± 3.12		439 (57.2%)		
Pregnancy intent, n(%)			0.790			0.426			0.296				0.636	
Planned	6.33 ± 2.90	153 (88.4%)		6.12 ± 2.96	176 (88.4%)		7.16 ± 3.20	362 (91.4%)		6.71 ± 3.10		691 (90.0%)		
Unplanned	6.15 ± 2.82	20 (11.6%)		6.83 ± 4.05	23 (11.6%)		6.56 ± 3.12	34 (8.6%)		6.53 ± 3.32		77 (10.0%)		
Pregnancy period, n(%)			-			-			-				< 0.001	
First trimester (≤ 13 weeks)	-	-		-	-		-	-		6.31 ± 2.88		173 (22.5%)		
Second trimester (14–27 weeks)	-	-		-	-		-	-		6.20 ± 3.10		199 (25.9%)		
Third trimester (≥ 28 weeks)	-	-		-	-		-	-		7.11 ± 3.19		396 (51.6%)		

### The relationship between fear of childbirth and sleep quality

**Fig. 1:**
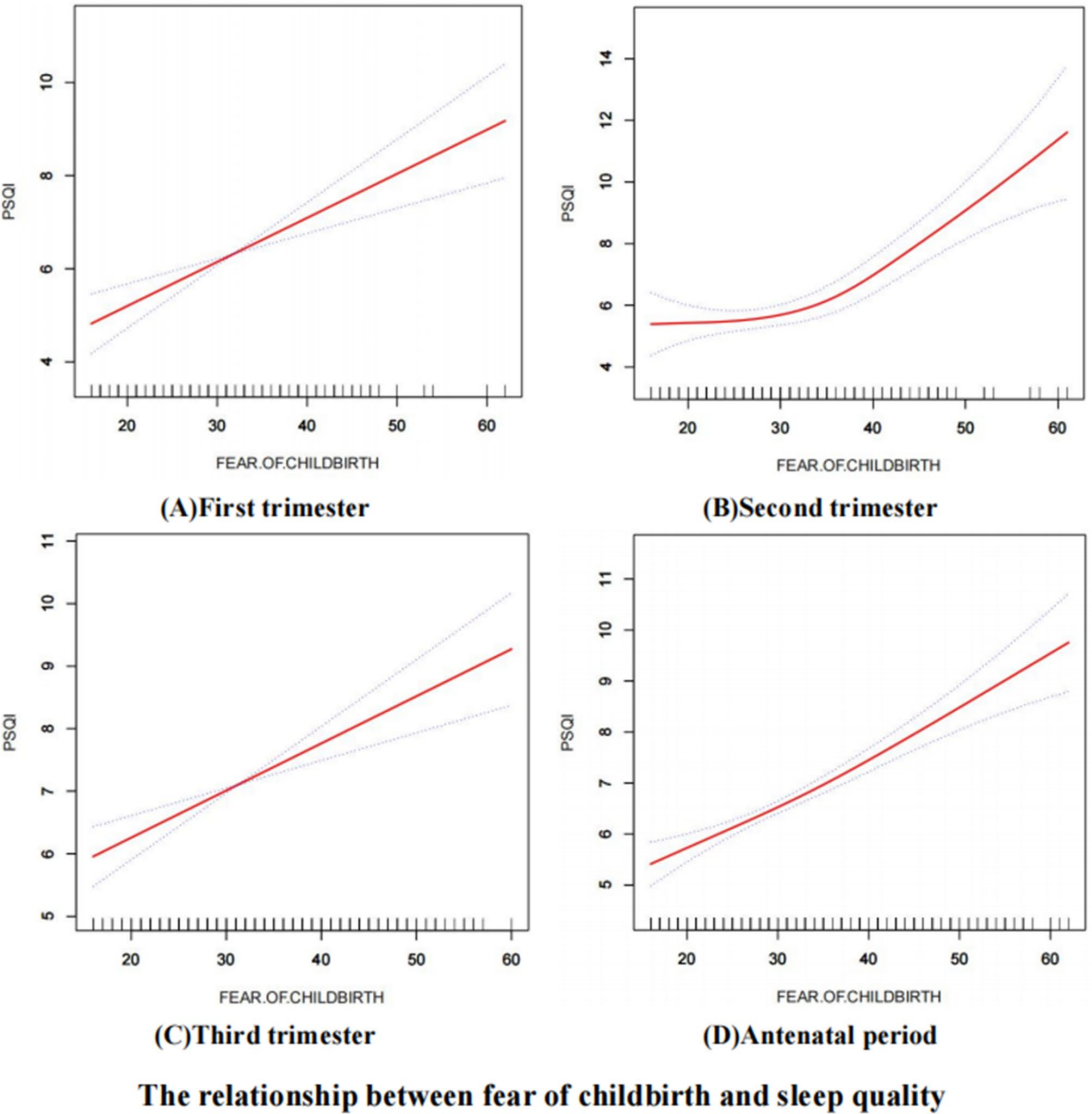
The smooth fitting curves of fear of childbirth and sleep quality

The binary logistic regression analysis revealed that fear of childbirth significantly affected sleep quality in all trimesters and the overall antenatal period, as represented in Table 2. Pregnant women exhibiting elevated levels of fear of childbirth were more likely to experience suboptimal sleep quality, which can be attributed to heightened sleep disturbances stemming from anxiety and concerns related to childbirth. Besides, the results of the generalized additive model analysis (Fig. 1) showed that fear of childbirth had a linear relationship with sleep quality in the first trimester and third trimester, while this association became nonlinear in the second trimester and antenatal period.

**Table 2:** The relationship between fear of childbirth and sleep quality (category variable)

Variable	*beta*	*SE*	*P value*	*OR value*	*LLCI*	*ULCI*
Model 1						
Constant	-2.911	0.620	< 0.001	0.054	-	-
Fear of childbirth (first trimester)	0.064	0.018	< 0.001	1.066	1.030	1.104
Model 2						
Constant	-3.610	0.612	< 0.001	0.027	-	-
Fear of childbirth (second trimester)	0.081	0.018	< 0.001	1.084	1.047	1.122
Model 3						
Constant	-1.513	0.354	< 0.001	0.220	-	-
Fear of childbirth (third trimester)	0.037	0.011	0.001	1.038	1.016	1.060
Model 4						
Constant	-2.265	0.270	< 0.001	0.104	-	-
Fear of childbirth (antenatal period)	0.052	0.008	< 0.001	1.053	1.037	1.070

### The meditation analysis

The first factor accounted for 24.47% (first trimester), 28.48% (second trimester), 27.34% (third trimester), and 26.73% (antenatal period) of the total variances and the common method bias was negligible. In the first trimester, PSQI showed a positive correlation with fear of childbirth (*r* = 0.330, *P* < 0.01) and psychological distress (*r* = 0.453, *P* < 0.01), while PSQI was negatively associated with resilience (*r*=-0.320, *P* < 0.01). Similarly, the associations among fear of childbirth, psychological distress, resilience, and PSQI were significant in the second trimester, third trimester, and antenatal period. Other correlation-related information is summarized in Table 3.

**Table 3:** Correlations between variables

		Fear of childbirth	Psychological distress	Resilience	PSQI score	
Model 1 (first trimester)						
Fear of childbirth		1				
Psychological distress		0.396**	1			
Resilience		-0.120**	-0.374**	1		
PSQI score		0.330**	0.453**	-0.320**	1	
Model 2 (second trimester)						
Fear of childbirth		1				
Psychological distress		0.445**	1			
Resilience		-0.316**	-0.559**	1		
PSQI score		0.376**	0.364**	-0.255**	1	
Model 3 (third trimester)						
Fear of childbirth		1				
Psychological distress		0.500**	1			
Resilience		-0.295**	-0.535**	1		
PSQI score		0.232**	0.391**	-0.175**	1	
Model 4 (antenatal period)						
Fear of childbirth		1				
Psychological distress		0.461**	1			
Resilience		-0.262**	-0.504**	1		
PSQI score		0.287**	0.394**	-0.226**	1	

Note. ** Correlation is significant at the 0.01 level (2-tailed)

The univariate analyses revealed that employment and income during the second trimester, age and academic degree during the third trimester, and age and pregnancy period during the antenatal phase had a significant impact on sleep quality. Consequently, these factors were incorporated into the mediated moderation model as confounding variables. In Table 4, the significant mediation role of psychological distress between fear of childbirth and sleep quality was recognized, including first trimester (*β* = 0.044, *95%CI*:0.022, 0.071), second trimester (*β* = 0.029, *95%CI*:0.009, 0.056), third trimester (*β* = 0.064, *95%CI*:0.046, 0.088), and antenatal period (*β* = 0.050, *95% CI*:0.037, 0.063).

**Table 4:** Mediation analysis results for the relationship between fear of childbirth and sleep quality

	*Effect size*	*SE*		*LLCI*	*ULCI*	
Model 1 (first trimester)						
Indirect effect	0.044	0.013		0.022	0.071	
Direct effect	0.051	0.021		0.010	0.093	
Total effect	0.095	0.021		0.054	0.136	
Model 2 (second trimester)						
Indirect effect	0.029	0.012		0.009	0.056	
Direct effect	0.080	0.023		0.036	0.125	
Total effect	0.109	0.021		0.068	0.151	
Model 3 (third trimester)						
Indirect effect	0.064	0.011		0.045	0.088	
Direct effect	0.012	0.017		-0.022	0.046	
Total effect	0.076	0.016		0.045	0.108	
Model 4 (antenatal period)						
Indirect effect	0.050	0.007		0.037	0.063	
Direct effect	0.045	0.012		0.022	0.068	
Total effect	0.095	0.011		0.073	0.116	

Moreover, in model 1, 95%CI of indirect effect (0.022, 0.071), direct effect (0.010, 0.093), and total effect (0.054, 0.136) implied psychological distress partially significantly mediated the relationship between fear of childbirth and sleep quality, with a mediating effect of 46.31%. Likewise, the mediating effects of psychological distress between fear of childbirth and sleep quality were 26.61% and 52.63% in the second trimester and antenatal period, respectively (model 2 and model 4). However, in model 3, 95%CI of the direct effect contained 0, so psychological distress fully mediated the relationship between fear of childbirth and sleep quality. The full details of the mediation analyses are presented in Supplementary Tables 1–4.

### The moderation role of resilience

**Fig. 2:**
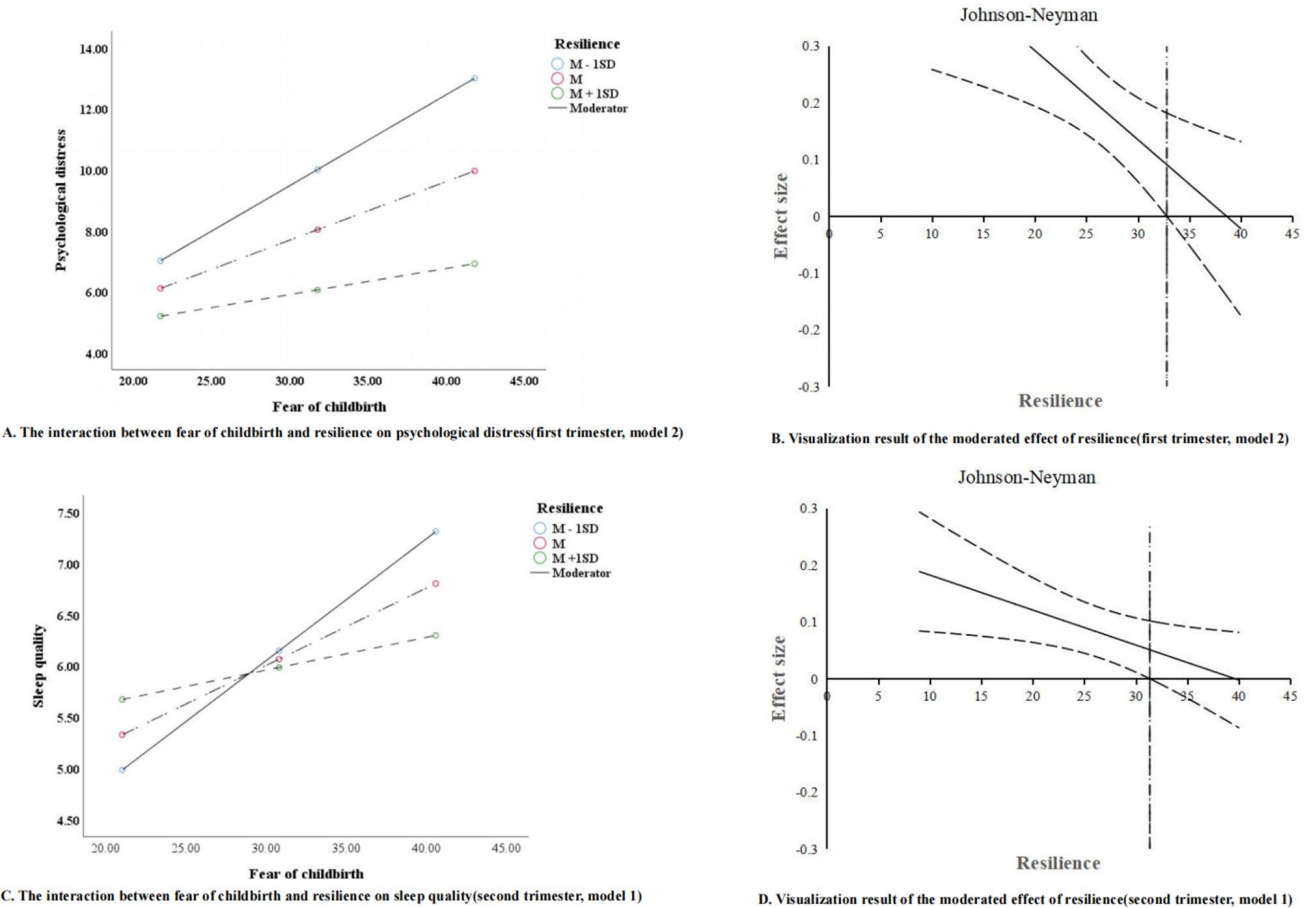
Simple slopes test and Johnson-Neyman test (First trimester and second trimester)

In Supplementary Table 5, the moderating effects of resilience were not significant in model 1 (*β*=-0.003, *P* = 0.306, *95%CI*:-0.009, 0.004) and model 3 (*β*=-0.002, *P* = 0.788, *95%CI*:-0.014, 0.011). However, in model 2, the interaction of fear of childbirth and resilience was significant (*β*=-0.016, *P* = 0.004, *95%CI*:-0.026, -0.005), signifying that resilience did moderate the relationship between fear of childbirth and psychological distress. Simple slope tests (Fig. 2-A) showed that the effect of fear of childbirth on psychological distress was statistically significant at a low level of resilience (*β* = 0.298, *95%CI*:0.197, 0.400) and a moderate level of resilience (*β* = 0.192, *95%CI*:0.126, 0.258); The effect was not significant for those with high resilience (*β* = 0.086, *95%CI*:-0.008, 0.179). Then, the Johnson-Neyman test (Fig. 2-B) indicated that there was a positive correlation between fear of childbirth and psychological distress when resilience was below 32.814, whereas the correlation gradually weakened and eventually disappeared as the value exceeded this threshold (*95%CI* of conditioned effect value of fear of childbirth on sleep quality included zero).

In Supplementary Table 6, the significant moderation effect of resilience (model 1) was recognized and visualized in the simple slopes test (Fig. 2-C), suggesting that resilience significantly moderated the relationship between fear of childbirth and sleep quality. Also, the boundary value of the moderation effect of resilience is 31.356 (Fig. 2-D). Nevertheless, the moderation effects of resilience were not significant in model 2 (*β*=-0.002, *P* = 0.570, *95%CI*:0.010, 0.005) and model 3 (*β*=-0.003, *P* = 0.564, *95%CI*:0.014, 0.008).

In Supplementary Table 7, the interaction terms in the path from fear of childbirth to sleep quality (model 1, *β*=-0.004, *P* = 0.100, *95%CI*:-0.008, 0.001), fear of childbirth to psychological distress (model 2, *β*=-0.003, *P* = 0.381, *95%CI*:-0.008, 0.003), and psychological distress to sleep quality (model 3, *β*=-0.003, *P* = 0.519, *95%CI*:-0.011, 0.006) were not significant.

**Fig. 3:**
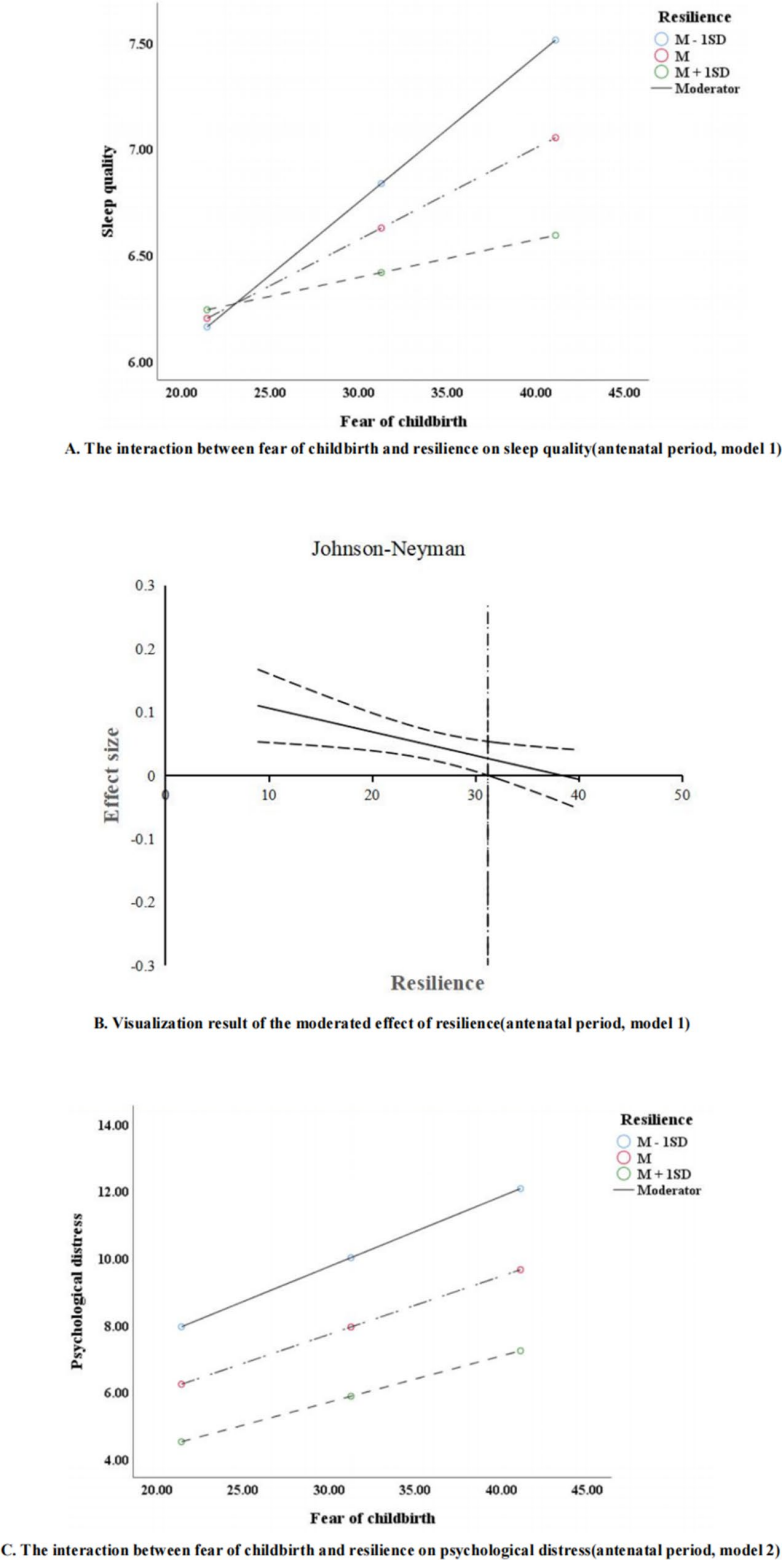
Simple slopes test and Johnson-Neyman test (Antenatal period)

In Supplementary Table 8, resilience acted as a significant moderator in the direct connection between fear of childbirth and sleep quality (model 1, *β*=-0.004, *P* = 0.014, *95%CI*:-0.007, -0.001), and the association between fear of childbirth and psychological distress (model 2, *β*=-0.004, *P* = 0.014, *95%CI*:-0.009, -0.001). However, the interaction between psychological distress and resilience was not significant (model 3, *β*=-0.003, *P* = 0.371, *95%CI*:-0.009, -0.003). Then, Fig. 3-A illustrates that the relationship between fear of childbirth and sleep quality strengthens in the case of low resilience (*β* = 0.069, *95%CI*:0.039, 0.095), as compared with moderate resilience (*β* = 0.043, *95%CI*:0.021, 0.066) and high resilience (*β* = 0.018, *95%CI*:-0.013, 0.049). Similarly, as shown in Fig. 3-C, compared to moderate resilience (*β* = 0.174, *95%CI*:0.145, 0.203) and high resilience (*β* = 0.138, *95%CI*:0.096, 0.180), the association between fear of childbirth and psychological distress was strengthened in case of low resilience (*β* = 0.210, *95%CI*:0.171, 0.249). Additionally, the Johnson-Neyman test (Fig. 3-B) indicated the relationship of fear of childbirth was more strongly related to sleep quality for those with a resilience score of 31.211 or less.

## Discussion

The current investigation aimed to examine the relationship between fear of childbirth and sleep quality while considering the potential mediating role of psychological distress and the moderating role of resilience. Findings substantiated the proposed associations and enriched our comprehension of these variables across different trimesters. First, as expected, fear of childbirth was negatively related to sleep quality, which was in line with previous studies [40–42]. A high level of fear of childbirth can lead to severe anxiety and fear in pregnant women. In this state, it can cause restlessness, sleeplessness, and lack of sleep time at night, resulting in poor sleep quality. Emerging studies indicated that cognitive-behavioral training and psychoeducation seemed to be effective in reducing fear of childbirth and improving sleep quality among pregnant women [43, 44]. These interventions can be adapted locally and applied to improve pregnant women’s physical and mental health worldwide.

Second, a non-linear association between fear of childbirth and sleep quality was identified in the second trimester and antenatal period, which had not been fully explored in previous research [42], contributing valuable insights to the existing fear of childbirth-sleep quality literature. The curvilinear relationship suggested that heterogeneity still exists within the different trimesters, and future studies could be enriched by conducting latent profile analysis or qualitative studies on pregnant women in both the second trimester and antenatal period.

Third, the mediation role of psychological distress was confirmed between fear of childbirth and sleep quality in all trimesters and the overall antenatal period, suggesting that fear of childbirth could have an indirect effect on sleep quality through psychological distress. Prior research supports the current finding by showing that fear of childbirth can lead to the development of psychological distress [18, 45], which subsequently has detrimental effects on the sleep quality of pregnant women [46, 47]. Anxiety/depression reduction interventions (i.e., mindfulness) could be developed and utilized in this population to improve their sleep quality. For example, interpersonal psychotherapy has been confirmed to be effective in preventing and treating psychological distress during pregnancy and postpartum [48–50], which could also be adapted to handle the fear of childbirth-induced sleep problems. In addition, the mediating effect of psychological distress was strongest in the third trimester, so future studies may focus on anxiety-depression interventions in this period to achieve a better effect.

Fourth, resilience did play a significant moderation role in fear of childbirth, psychological distress, and sleep quality. In other words, fear of childbirth would have a weaker association with sleep quality as resilience increased, especially among pregnant women with resilience scores greater than 31 in the second trimester and antenatal period. Besides, the correlation between fear of childbirth and psychological distress in those with high resilience levels was weaker than in those with low resilience levels (first trimester and antenatal period). This may be attributed to the fact that resilience plays a significant role in buffering against the fear of childbirth and psychological distress among pregnant women [10, 51]. Compared to those with low levels of resilience, pregnant women with high resilience levels better handle with fear of childbirth-induced psychological distress and maintain better sleep quality [52–54]. Furthermore, resilience-enhancing interventions have demonstrated positive effects on resilience and the preservation of mental well-being among pregnant women [55, 56]. These successful programs could also be adapted for wider application to more pregnant women to improve their resilience, so more research should be warranted in the future. Based on these findings, pregnant women with high levels of fear of childbirth, psychological distress, and low resilience levels may be vulnerable to sleep disturbance or disorder and should be given more attention.

To be brief, the current study contributes to our understanding of the relationships among fear of childbirth, psychological distress, resilience, and sleep quality based on a sample of Chinese pregnant women. It demonstrates that (1) fear of childbirth is negatively correlated with sleep quality; (2) The association between fear of childbirth and sleep quality is significantly mediated by psychological distress and moderated by resilience.

### Strengths, limitations, and further Scopes

The primary strength of this study lies in the implementation of stratified analyses, which effectively identified variations across trimesters, enhanced result precision, and offered valuable insights for targeted interventions and support. Moreover, the examination of the mediating influence of psychological distress and the moderating effect of resilience contributed to a comprehensive comprehension of the intricate relationship among these factors. However, several limitations should be contemplated. First, the incidence of fear of childbirth among pregnant women in China is higher than those in other countries. Therefore, the findings and conclusions of the present study may not be generalized to pregnant women of different backgrounds. Besides, a causal relationship couldn’t be well settled because of the cross-sectional nature of this study, and a fixed cohort study with 4 waves of follow-up during the entire gestation period ought to be further performed to validate these findings. Third, in the moderated mediation model, a few potential confounders, i.e., social support, intimate partner violence, etc., are not considered because of the heavy scale burden, which will affect the association estimation.

## Conclusions

Fear of childbirth, psychological distress, and resilience are three important factors affecting sleep quality in Chinese pregnant women.

### Electronic supplementary material

Below is the link to the electronic supplementary material.

**Supplementary Material 1:** The results of mediated moderated analysis

## Data Availability

The data that support the findings of this study are available on request from the corresponding author. The data are not publicly available due to privacy or ethical restrictions.
