# Supplementary material for: Fear of childbirth and sleep quality among pregnant women: a generalized additive model and moderated mediation analysis
Source: BMC Psychiatry. 2023 Dec 11;23:931. doi: 10.1186/s12888-023-05435-y (PMC10712172; doi:10.1186/s12888-023-05435-y)
Supplement: Supplementary file 1 — Supplementary Material 1: The results of mediated moderated analysis [file 12888_2023_5435_MOESM1_ESM.docx]

| **Supplementary Table 1 The mediating effect of psychological distress on sleep quality (first trimester)** | | | | | | | |
| --- | --- | --- | --- | --- | --- | --- | --- |
| **Outcome**  **variable** | **Predictor variable** | **Biased regression coefficient** | | | | | |
|  |  | ***beta*** | ***SE*** | ***t*** | ***P*** | ***LLCI*** | ***ULCI*** |
| Sleep quality |  |  |  |  |  |  |  |
|  | Constant | 3.302 | 0.689 | 4.789 | <0.001 | 1.941 | 4.662 |
|  | Fear of childbirth | 0.095 | 0.021 | 4.578 | <0.001 | 0.054 | 0.136 |
| Psychological distress |  |  |  |  |  |  |  |
|  | Constant | 1.671 | 1.207 | 1.384 | 0.168 | -0.713 | 4.054 |
|  | Fear of childbirth | 0.205 | 0.036 | 5.642 | <0.001 | 0.133 | 0.276 |
| Sleep quality |  |  |  |  |  |  |  |
|  | Constant | 2.947 | 0.645 | 4.565 | <0.001 | 1.673 | 4.221 |
|  | Fear of childbirth | 0.051 | 0.021 | 2.446 | 0.016 | 0.010 | 0.093 |
|  | Psychological distress | 0.212 | 0.041 | 5.226 | <0.001 | 0.132 | 0.293 |

| **Supplementary Table 2 The mediating effect of psychological distress on sleep quality (second trimester)** | | | | | | | |
| --- | --- | --- | --- | --- | --- | --- | --- |
| **Outcome**  **variable** | **Predictor variable** | **Biased regression coefficient** | | | | | |
|  |  | ***beta*** | ***SE*** | ***t*** | ***P*** | ***LLCI*** | ***ULCI*** |
| Sleep quality |  |  |  |  |  |  |  |
|  | Constant | 3.292 | 0.808 | 4.075 | <0.001 | 1.699 | 4.885 |
|  | Employment | 0.822 | 0.463 | 1.774 | 0.078 | -0.921 | 1.736 |
|  | Income | -0.922 | 0.474 | -1.946 | 0.053 | -1.856 | 0.013 |
|  | Fear of childbirth | 0.109 | 0.021 | 5.248 | <0.001 | 0.068 | 0.151 |
| Psychological distress |  |  |  |  |  |  |  |
|  | Constant | 3.140 | 1.237 | 2.538 | 0.012 | 0.700 | 5.581 |
|  | Employment | -0.266 | 0.710 | -0.375 | 0.708 | -1.666 | 1.134 |
|  | Income | -2.345 | 0.726 | -3.231 | 0.001 | -3.776 | -0.913 |
|  | Fear of childbirth | 0.211 | 0.032 | 6.604 | <0.001 | 0.148 | 0.274 |
| Sleep quality |  |  |  |  |  |  |  |
|  | Constant | 2.855 | 0.804 | 3.550 | <0.001 | 1.269 | 4.441 |
|  | Employment | 0.859 | 0.454 | 1.892 | 0.060 | -0.037 | 1.754 |
|  | Income | -0.596 | 0.476 | -1.250 | 0.213 | -1.535 | 0.344 |
|  | Fear of childbirth | 0.080 | 0.023 | 3.550 | <0.001 | 0.036 | 0.125 |
|  | Psychological distress | 0.139 | 0.046 | 3.039 | 0.003 | 0.049 | 0.230 |

| **Supplementary Table 3 The mediating effect of psychological distress on sleep quality (third trimester)** | | | | | | | |
| --- | --- | --- | --- | --- | --- | --- | --- |
| **Outcome**  **variable** | **Predictor variable** | **Biased regression coefficient** | | | | | |
|  |  | ***beta*** | ***SE*** | ***t*** | ***P*** | ***LLCI*** | ***ULCI*** |
| Sleep quality |  |  |  |  |  |  |  |
|  | Constant | 1.359 | 1.183 | 1.148 | 0.252 | -0.968 | 3.686 |
|  | Age | 0.107 | 0.036 | 2.982 | 0.003 | 0.036 | 0.177 |
|  | Academic degree | 0.225 | 0.195 | 1.155 | 0.249 | -0.158 | 0.608 |
|  | Fear of childbirth | 0.076 | 0.016 | 4.814 | <0.001 | 0.045 | 0.108 |
| Psychological distress |  |  |  |  |  |  |  |
|  | Constant | 1.904 | 1.602 | 1.189 | 0.235 | -1.245 | 5.053 |
|  | Age | -0.035 | 0.049 | -0.724 | 0.470 | -0.130 | 0.060 |
|  | Academic degree | -0.609 | 0.264 | -2.309 | 0.021 | -1.127 | -0.091 |
|  | Fear of childbirth | 0.249 | 0.021 | 11.630 | <0.001 | 0.207 | 0.292 |
| Sleep quality |  |  |  |  |  |  |  |
|  | Constant | 0.867 | 1.112 | 0.779 | 0.436 | -1.320 | 3.053 |
|  | Age | 0.116 | 0.034 | 3.447 | <0.001 | 0.050 | 0.182 |
|  | Academic degree | 0.382 | 0.184 | 2.079 | 0.038 | 0.021 | 0.744 |
|  | Fear of childbirth | 0.012 | 0.017 | 0.684 | 0.495 | -0.022 | 0.046 |
|  | Psychological distress | 0.259 | 0.035 | 7.388 | <0.001 | 0.190 | 0.327 |

| **Supplementary Table 4 The mediating effect of psychological distress on sleep quality (antenatal period)** | | | | | | | |
| --- | --- | --- | --- | --- | --- | --- | --- |
| **Outcome**  **variable** | **Predictor variable** | **Biased regression coefficient** | | | | | |
|  |  | ***beta*** | ***SE*** | ***t*** | ***P*** | ***LLCI*** | ***ULCI*** |
| Sleep quality |  |  |  |  |  |  |  |
|  | Constant | 1.023 | 0.807 | 1.267 | 0.205 | -0.561 | 2.606 |
|  | Age | 0.073 | 0.024 | 3.124 | 0.002 | 0.027 | 0.119 |
|  | Pregnancy period | 0.444 | 0.132 | 3.124 | <0.001 | 0.185 | 0.703 |
|  | Fear of childbirth | 0.095 | 0.011 | 8.686 | <0.001 | 0.073 | 0.116 |
| Psychological distress |  |  |  |  |  |  |  |
|  | Constant | 2.780 | 1.193 | 2.347 | 0.019 | 0.458 | 5.141 |
|  | Age | -0.068 | 0.035 | -1.946 | 0.052 | -0.136 | 0.001 |
|  | Pregnancy period | 0.055 | 0.195 | 0.282 | 0.778 | -0.328 | 0.438 |
|  | Fear of childbirth | 0.228 | 0.016 | 14.199 | <0.001 | 0.197 | 0.260 |
| Sleep quality |  |  |  |  |  |  |  |
|  | Constant | 0.416 | 0.768 | 0.541 | 0.588 | -1.091 | 1.922 |
|  | Age | 0.088 | 0.022 | 3.944 | <0.001 | 0.044 | 0.132 |
|  | Pregnancy period | 0.432 | 0.125 | 3.455 | <0.001 | 0.187 | 0.678 |
|  | Fear of childbirth | 0.045 | 0.012 | 3.880 | <0.001 | 0.022 | 0.068 |
|  | Psychological distress | 0.217 | 0.023 | 9.348 | <0.001 | 0.171 | 0.262 |

| **Supplementary Table 5 The moderating effect of resilience between fear of childbirth, psychological distress, and sleep quality (first trimester)** | | | | | | |
| --- | --- | --- | --- | --- | --- | --- |
| **Variable** | ***Estimate*** | ***SE*** | ***t*** | ***P*** | ***LLCI*** | ***ULCI*** |
| Model 1 fear of childbirth → sleep quality |  |  |  |  |  |  |
| Constant | 2.760 | 2.649 | 1.042 | 0.299 | -2.468 | 7.989 |
| Fear of childbirth | 0.141 | 0.088 | 1.598 | 0.112 | -0.033 | 0.316 |
| Psychological distress | 0.164 | 0.044 | 3.726 | <0.001 | 0.077 | 0.250 |
| Resilience | 0.015 | 0.094 | 0.159 | 0.874 | -0.171 | 0.201 |
| Fear of childbirth × Resilience | -0.003 | 0.003 | -1.026 | 0.306 | -0.009 | 0.003 |
| Model 2 fear of childbirth → psychological distress |  |  |  |  |  |  |
| Constant | -3.507 | 4.630 | -0.757 | 0.450 | -12.647 | 5.633 |
| Fear of childbirth | 0.605 | 0.148 | 4.104 | <0.001 | 0.314 | 0.896 |
| Resilience | 0.207 | 0.164 | 1.258 | 0.210 | -0.118 | 0.531 |
| Fear of childbirth × Resilience | -0.016 | 0.005 | -2.933 | 0.004 | -0.026 | -0.005 |
| Model 3 psychological distress → sleep quality |  |  |  |  |  |  |
| Constant | 4.924 | 1.591 | 3.095 | 0.002 | 1.783 | 8.604 |
| Fear of childbirth | 0.053 | 0.021 | 2.570 | 0.011 | 0.012 | 0.094 |
| Psychological distress | 0.214 | 0.156 | 1.373 | 0.172 | -0/094 | 0.521 |
| Resilience | -0.065 | 0.053 | -1.228 | 0.221 | -0.169 | 0.040 |
| Psychological distress × Resilience | -0.002 | 0.006 | -0.269 | 0.788 | -0.014 | 0.011 |

| **Supplementary Table 6 The moderating effect of resilience between fear of childbirth, psychological distress, and sleep quality (second trimester)** | | | | | | |
| --- | --- | --- | --- | --- | --- | --- |
| **Variable** | ***Estimate*** | ***SE*** | ***t*** | ***P*** | ***LLCI*** | ***ULCI*** |
| Model 1 fear of childbirth → sleep quality |  |  |  |  |  |  |
| Constant | -1.965 | 2.632 | -0.747 | 0.456 | -7.156 | 3.225 |
| Employment | 0.809 | 0.452 | 1.789 | 0.075 | -0.083 | 1.701 |
| Income | -0.548 | 0.477 | -1.149 | 0.252 | -1.490 | 0.393 |
| Fear of childbirth | 0.244 | 0.076 | 3.211 | 0.002 | 0.094 | 0.394 |
| Psychological distress | 0.129 | 0.051 | 2.518 | 0.013 | 0.028 | 0.231 |
| Resilience | 0.178 | 0.089 | 2.010 | 0.046 | -0.012 | -0.001 |
| Fear of childbirth × Resilience | -0.006 | 0.003 | -2.264 | 0.025 | -0.012 | -0.001 |
| Model 2 fear of childbirth → psychological distress |  |  |  |  |  |  |
| Constant | 10.987 | 3.601 | 3.051 | 0.003 | 3.884 | 18.090 |
| Employment | -0.575 | 0.633 | -0.909 | 0.364 | -1.823 | 0.673 |
| Income | -1.224 | 0.663 | -1.846 | 0.066 | -2.532 | 0.084 |
| Fear of childbirth | 0.206 | 0.106 | 1.956 | 0.052 | -0.002 | 0.414 |
| Resilience | -0.244 | 0.123 | -1.979 | 0.049 | -0.487 | -0.001 |
| Fear of childbirth × Resilience | -0.002 | 0.004 | -0.569 | 0.570 | -0.010 | 0.005 |
| Model 3 psychological distress → sleep quality |  |  |  |  |  |  |
| Constant | 2.428 | 1.820 | 1.335 | 0.184 | -1.161 | 6.017 |
| Employment | 0.835 | 0.458 | 1.822 | 0.070 | -0.069 | 1.739 |
| Income | -0.560 | 0.485 | -1.155 | 0.249 | -1.515 | 0.396 |
| Fear of childbirth | 0.080 | 0.023 | 3.528 | <0.001 | 0.036 | 0.125 |
| Psychological distress | 0.213 | 0.146 | 1.458 | 0.147 | -0.075 | 0.501 |
| Resilience | 0.016 | 0.058 | 0.300 | 0.764 | -0.088 | 0.120 |
| Psychological distress × Resilience | -0.003 | 0.006 | -0.578 | 0.564 | -0.014 | 0.008 |

| **Supplementary Table 7 The moderating effect of resilience between fear of childbirth, psychological distress, and sleep quality (third trimester)** | | | | | | |
| --- | --- | --- | --- | --- | --- | --- |
| **Variable** | ***Estimate*** | ***SE*** | ***t*** | ***P*** | ***LLCI*** | ***ULCI*** |
| Model 1 fear of childbirth → sleep quality |  |  |  |  |  |  |
| Constant | -2.196 | 2.172 | -1.011 | 0.313 | -6.466 | 2.075 |
| Age | 0.114 | 0.034 | 3.366 | <0.001 | 0.047 | 0.180 |
| Academic degree | 0.381 | 0.184 | 2.069 | 0.039 | 0.019 | 0.743 |
| Fear of childbirth | 0.109 | 0.061 | 1.800 | 0.073 | -0.010 | 0.228 |
| Psychological distress | 0.261 | 0.039 | 6.618 | <0.001 | 0.183 | 0.338 |
| Resilience | 0.117 | 0.071 | 1.652 | 0.099 | -0.022 | 0.256 |
| Fear of childbirth × Resilience | -0.004 | 0.002 | -1.671 | 0.100 | -0.008 | 0.001 |
| Model 2 fear of childbirth → psychological distress |  |  |  |  |  |  |
| Constant | 8.121 | 2.764 | 2.938 | 0.004 | 2.687 | 13.555 |
| Age | 0.018 | 0.044 | 0.420 | 0.674 | -0.067 | 0.104 |
| Academic degree | -0.320 | 0.237 | -1.353 | 0.177 | -0.785 | 0.145 |
| Fear of childbirth | 0.253 | 0.077 | 3.293 | 0.001 | 0.102 | 0.404 |
| Resilience | -0.229 | 0.090 | -2.536 | 0.012 | -0.407 | -0.052 |
| Fear of childbirth × Resilience | -0.003 | 0.003 | -0.877 | 0.381 | -0.008 | 0.003 |
| Model 3 psychological distress → sleep quality |  |  |  |  |  |  |
| Constant | 0.132 | 1.575 | 0.084 | 0.933 | -2.965 | 3.229 |
| Age | 0.116 | 0.034 | 3.411 | <0.001 | 0.049 | 0.183 |
| Academic degree | 0.380 | 0.185 | 2.055 | 0.041 | 0.016 | 0.743 |
| Fear of childbirth | 0.013 | 0.017 | 0.742 | 0.459 | -0.021 | 0.047 |
| Psychological distress | 0.330 | 0.110 | 3.004 | 0.003 | 0.114 | 0.545 |
| Resilience | 0.025 | 0.039 | 0.655 | 0.513 | -0.051 | 0.101 |
| Psychological distress × Resilience | -0.003 | 0.004 | -0.646 | 0.519 | -0.011 | 0.006 |

| **Supplementary Table 8 The moderating effect of resilience between fear of childbirth, psychological distress, and sleep quality (antenatal period)** | | | | | | |
| --- | --- | --- | --- | --- | --- | --- |
| **Variable** | ***Estimate*** | ***SE*** | ***t*** | ***P*** | ***LLCI*** | ***ULCI*** |
| Model 1 fear of childbirth → sleep quality |  |  |  |  |  |  |
| Constant | -1.732 | 1.465 | -1.182 | 0.238 | -4.608 | 1.145 |
| Age | 0.089 | 0.022 | 3.951 | <0.001 | 0.045 | 0.133 |
| Pregnancy period | 0.417 | 0.125 | 3.342 | <0.001 | 0.172 | 0.662 |
| Fear of childbirth | 0.144 | 0.042 | 3.417 | <0.001 | 0.061 | 0.226 |
| Psychological distress | 0.195 | 0.026 | 7.550 | <0.001 | 0.145 | 0.246 |
| Resilience | 0.086 | 0.048 | 1.790 | 0.074 | -0.008 | 0.181 |
| Fear of childbirth × Resilience | -0.004 | 0.002 | -2.454 | 0.014 | -0.007 | -0.001 |
| Model 2 fear of childbirth → psychological distress |  |  |  |  |  |  |
| Constant | 6.565 | 2.038 | 3.221 | 0.001 | 2.564 | 10.567 |
| Age | -0.011 | 0.031 | -0.356 | 0.722 | -0.073 | 0.050 |
| Pregnancy period | 0.024 | 0.175 | 0.137 | 0.891 | -0.319 | 0.367 |
| Fear of childbirth | 0.315 | 0.058 | 5.456 | <0.001 | 0.202 | 0.429 |
| Resilience | -0.140 | 0.067 | -2.085 | 0.037 | -0.272 | -0.008 |
| Fear of childbirth × Resilience | -0.005 | 0.002 | -2.477 | 0.014 | -0.009 | -0.001 |
| Model 3 psychological distress → sleep quality |  |  |  |  |  |  |
| Constant | 0.590 | 1.069 | 0.552 | 0.581 | -1.509 | 2.689 |
| Age | 0.092 | 0.023 | 4.090 | <0.001 | 0.048 | 0.136 |
| Pregnancy period | 0.429 | 0.125 | 3.427 | <0.001 | 0.183 | 0.674 |
| Fear of childbirth | 0.045 | 0.012 | 3.885 | <0.001 | 0.022 | 0.068 |
| Psychological distress | 0.265 | 0.077 | 3.471 | 0.001 | 0.115 | 0.416 |
| Resilience | -0.006 | 0.027 | -0.212 | 0.832 | -0.059 | 0.047 |
| Psychological distress × Resilience | -0.003 | 0.003 | -0.895 | 0.371 | -0.009 | 0.003 |
